# Isolation and Infrared Spectroscopic Characterization of Hemibonded Water Dimer Cation in Superfluid Helium Nanodroplets

**DOI:** 10.1021/acs.jpclett.3c02150

**Published:** 2023-09-06

**Authors:** Arisa Iguchi, Amandeep Singh, Stefan Bergmeister, Andrew A. Azhagesan, Kenta Mizuse, Asuka Fujii, Hajime Tanuma, Toshiyuki Azuma, Paul Scheier, Susumu Kuma, Andrey F. Vilesov

**Affiliations:** † Department of Physics, Tokyo Metropolitan University, Tokyo 192-0397, Japan; ‡ Atomic, Molecular, and Optical Physics Laboratory, RIKEN, Saitama 351-0198, Japan; § Department of Chemistry, University of Southern California, Los Angeles, California 90089, United States; ∥ Institut für Ionenphysik und Angewandte Physik, Universität Innsbruck, A-6020 Innsbruck, Austria; ⊥ Department of Computer Science, University of Southern California, Los Angeles, California 90089, United States; # Department of Chemistry, School of Science, Kitasato University, Sagamihara, Kanagawa 252-0373, Japan; % Department of Chemistry, Graduate School of Science, Tohoku University, Sendai 980-8578, Japan

## Abstract

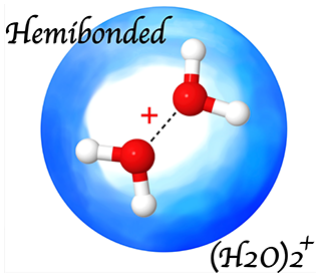

The structure of the minimum unit of the radical cationic water clusters, the (H_2_O)_2_^+^ dimer, has attracted much attention because of its importance for the radiation chemistry of water. Previous spectroscopic studies indicated that the dimers have a proton-transferred structure (H_3_O^+^·OH), though the alternate metastable hemibonded structure (H_2_O·OH_2_)^+^ was also predicted based on theoretical calculations. Here, we produce (H_2_O)_2_^+^ dimers in superfluid helium nanodroplets and study their infrared spectra in the range of OH stretching vibrations. The observed spectra indicate the coexistence of the two structures in the droplets, supported by density functional theory calculations. This is the first spectroscopic identification of the hemibonded isomer of water radical cation dimers. The observation of the higher-energy isomer reveals efficient kinetic trapping for metastable ionic clusters due to the rapid cooling in helium droplets.

The interaction of a high-energy photon or particle with liquid water or aqueous solutions leads to the ionization of water. The resulting H_2_O^+^ ions and electrons (e^–^) trigger a rich manifold of secondary reactions.^1^ Because the ionization of water occurs in a wide range of chemical, biological, and astrochemical environments, it is of fundamental importance to understand the fate of H_2_O^+^ and e^–^ in the presence of neighboring water at the molecular level. The formation process and properties of the hydrated electron have been extensively studied primarily by ultrafast optical and photoelectron spectroscopy.^2−5^ It was assumed that H_2_O^+^ rapidly turns into the hydronium H_3_O^+^ ions via proton transfer from the neighboring H_2_O molecules.^1,6,7^ The attempts to observe this mechanism via spectroscopic technique were inconclusive because of the short lifetime of the H_2_O^+^ and overlap of the spectra of the cationic species with the strong absorption of the hydrated electrons.^8,9^ Recent ultrafast (<100 fs) X-ray spectroscopic and electron diffraction experiments provided the evidence of the OH radical and H_3_O^+^ cation pair.^6,7,10^ However, the incipient dynamics of the cations in water remains veiled.

Charged water clusters were widely used to elucidate the structures of excess charged species. In protonated water clusters, the motifs include H_3_O^+^ and H_5_O_2_^+^, depending on the cluster size.^11,12^ Gardenier et al.^13^ studied the radical cationic clusters (H_2_O)_2_^+^ by Ar-tagged predissociation spectroscopy. They concluded that (H_2_O)_2_^+^ predominantly has the proton-transferred (PT) structure, (H_3_O^+^·OH). Larger clusters also contain the H^+^(H_2_O)_*n*_OH motif.^14,15^ In these studies, the ionic clusters were obtained in a supersonic expansion at a relatively high temperature of about 100 K and experienced a large number of collisions along the expansion trajectory. Therefore, higher energy isomers, such as those containing the hemi motif, may relax to a lower energy PT configuration.

Quantum chemical calculations show that the (H_2_O)_*n*_^+^ cation has two key motifs.^16−20^ The first is the PT structure, containing a hydronium ion–OH radical pair (H_3_O^+^·OH). The other motif is the hemibonded (Hemi-) structure, in which the unpaired electron and excess charge are shared between the two H_2_O moieties, resulting in a 2 center–3 electron bond (H_2_O·OH_2_)^+^ with a bond order of 1/2. High-level computational studies predicted that the PT type would be more stable than the Hemi type,^16−19^ which is in agreement with the previous spectroscopic observation of the PT type clusters. However, calculations also found a substantial potential barrier between the PT and Hemi structures, indicating that a metastable Hemi-type structure can be formed upon rapid cooling and trapping. By comparison, in the sulfur-containing analogue of water, H_2_S, the Hemi-type motif, (H_2_S·SH_2_)^+^, has been identified in both the gas and condensed phases.^21,22^

The existence of the Hemi-type water dimer cation has been an important question in order to understand the mechanism of formation of a hydrogen bond network in ionized water. The Hemi dimer may provide an alternative to proton transfer for stabilization of an excess positive charge in the minimum network unit. Mass spectrometric studies on (H_2_O)_*n*_^+^ clusters have suggested the existence of the Hemi-type ions, where the appearance of H_2_O^+^ fragments upon collisions of (H_2_O)_2_^+^ with other species was interpreted as evidence of the Hemi dimers.^23,24^ In a related study involving H_2_S, however, the protonated fragments originate from the dissociation of the Hemi-type ion, (H_2_S·OHCH_3_)^+^, as a result of rearrangements during dissociation.^25^ Even with this related evidence, spectroscopic characterization is required to verify the existence of the Hemi-type water dimer cation.

Here we report an infrared (IR) spectroscopic investigation of the structure of (H_2_O)_2_^+^ created in helium droplets upon ionization of water dimers. The ions are produced in an ultracold (0.4 K) environment, and therefore the stabilization of higher energy isomers of (H_2_O)_2_^+^ may be facilitated. A detailed description of the experiments can be found elsewhere.^26−28^ Helium droplets are produced upon pulsed (180 μs) expansion of helium gas at 20 bar and at a temperature of 19 or 23 K. The droplets pass through a 2 mm skimmer and pick up several water molecules in a 44 cm long differentially pumped pickup chamber containing water vapor. The droplets pass through another differential pumping stage and enter the detection chamber. In this chamber, the droplets are ionized by electron impact, which yields (H_2_O)_2_^+^ embedded in helium droplets. The ionic droplets traverse into the ion range of a quadrupole mass spectrometer (QMS, Extrel MAX 500), where they are exposed to a pulsed IR laser beam. Vibrational excitation of the embedded (H_2_O)_2_^+^ causes the evaporation of the helium droplets and release of the bare (H_2_O)_2_^+^ cations, which are further mass-selected and detected by QMS. The output signal from an electron multiplier is amplified and recorded by an SR250 boxcar integrator with a typical gate width of 5–10 μs. IR spectra were measured by monitoring the yield of (H_2_O)_2_^+^ as a function of the IR wavenumber. During the described experiments, we installed an octupole ion guide collision cell (Extrel, model 815882) between the ionization and the IR interaction regions to enhance the evaporation of droplets. The addition of the ion guide ensures that the ionized droplets are optimally transmitted from the ionization region to the QMS. The droplets could also be reduced in diameter upon injecting an additional helium gas into the collision cell, which greatly increased the intensity of the measured spectrum.

The mid-IR laser pulses are produced by an Nd:YAG pumped OPO/OPA system with a line width of about 1 cm^–1^ (LaserVision, pulse duration ∼7 ns, pulse energy ∼4 mJ, repetition rate 20 Hz). The laser cabinet and path to the optical window of the experimental setup are purged by dry air or nitrogen gas to minimize the contribution from water absorption in the air. The laser wavenumber is calibrated by recording the photoacoustic spectra of methane and water in the range from 2720 to 3850 cm^–1^. The accuracy of the wavenumbers reported in this work is estimated to be 0.6 cm^–1^ from the standard deviation in the fitting of the calibration spectra.

Figure 1 shows the IR spectra measured when the mass spectrometer was tuned to *m*/*q* = 36 for the (H_2_O)_2_^+^ ions. The red trace (a) was measured with the nozzle at 23 K without the octupole ion guide. The black trace (b) was obtained in the measurements with the octupole ion guide filled with helium gas at a nominal pressure of 5.0 × 10^–5^ mbar and with the nozzle operating at 19 K. Figure 2 shows the pickup pressure dependences of the intensity of the 3610.8 cm^–1^ band under these two conditions. The results were fitted to the Poisson equation for the pickup process: *P*_*k*_(*z*) = *z*^*k*^ exp(−*z*)/*k*!, where *z* is the average number of the captured molecules and *k* is the number of water molecules captured per droplet.^29^*z* is proportional to the water pressure in the pickup chamber, the droplet’s cross section, and the length of the pickup chamber. The observed pressure dependences fit well with *k* = 2 at both nozzle temperatures, showing that the (H_2_O)_2_^+^ ions predominantly originate from the ionization of the (H_2_O)_2_ dimers. A significant deviation of the data points from the fitting curve is observed at high pickup pressure for the measurements at 19 K. This deviation likely corresponds to the signal produced from larger ionic clusters. The maximum of the signal at 19 K is achieved at the pickup pressure which is about a factor of 5 smaller than the corresponding pressure at 23 K. From the pickup pressure and the length of the pickup region, we estimated that the average droplets that give rise to the (H_2_O)_2_^+^ signal obtained at 19 and 23 K contain 2.0 × 10^5^ and 1.6 × 10^4^ He atoms and have radii of 13 and 5.6 nm, respectively.

**Figure 1:**
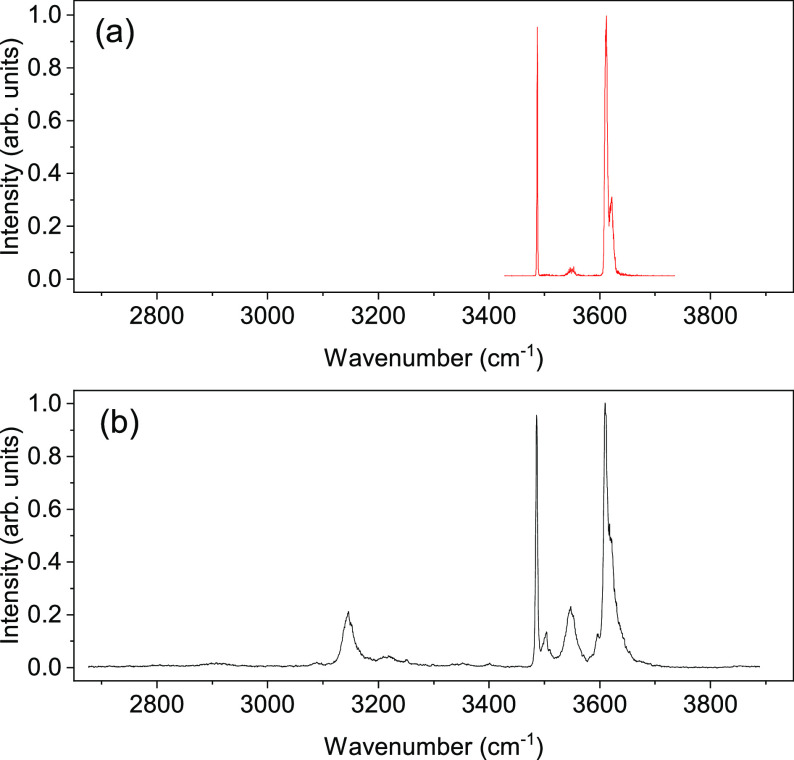
IR spectra of (H_2_O)_2_^+^ as measured at a mass channel of *m*/*q* = 36 in helium droplets (a) at a nozzle temperature of 23 K without the octupole ion guide and (b) at a nozzle temperature of 19 K with the octupole ion guide. The water pickup pressures were 2.0 × 10^–6^ and 1.2 × 10^–7^ mbar, respectively. Each spectrum was normalized to the peak intensity of the band at 3610.8 cm^–1^.

**Figure 2:**
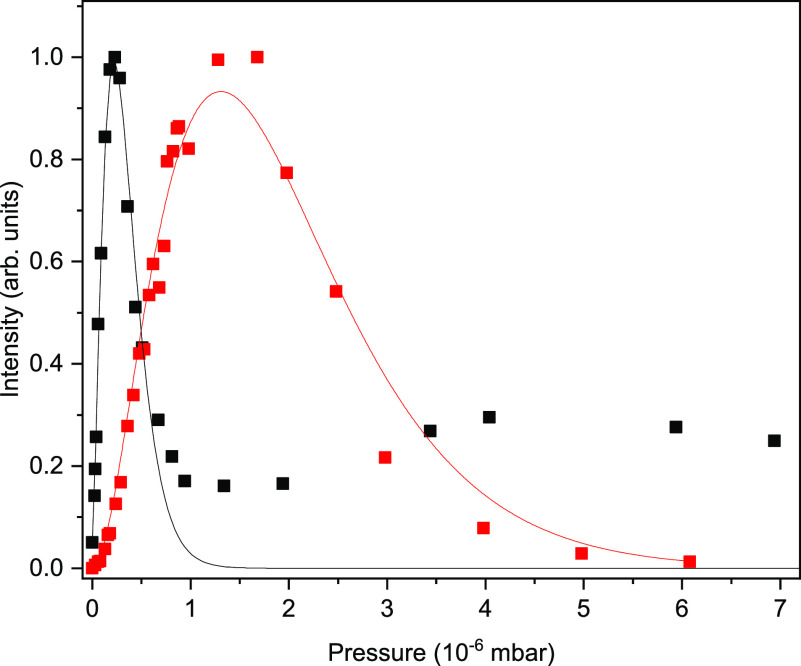
Water pickup pressure dependence of the intensity of the (H_2_O)_2_^+^ band at 3610.8 cm^–1^. The black trace and the red trace were obtained in the experiments performed at the nozzle temperature of 19 and 23 K, respectively. Each data set was normalized to 1 at the maximum. Solid curves represent the results of fitting to the Poisson equation (see text) with *k* = 2.

The spectra of (H_2_O)_2_^+^ in Figure 1 have strong peaks at 3610.8 and 3486.6 cm^–1^. In trace (a), the peak at 3610.8 cm^–1^ has a weaker companion at 3619.7 cm^–1^, which is seen more clearly as a shoulder in trace (b). Trace (b) shows another peak at 3503.8 cm^–1^. In both spectra, a weaker and broader peak is also found at around 3550 cm^–1^. While trace (a) spans a limited spectral range, trace (b) measured in a wider spectral range shows an additional band at 3146.1 cm^–1^. The trace (b) measured with the octupole collision cell has about a factor of ∼50 larger intensity. The comparison of both traces shows that the bands have different relative intensities. This effect is likely caused by the nonlinear laser pulse energy dependence^26,27^ of the ion signal, which makes broad bands in the trace (a) weaker. On the other hand, the intensities of the bands in trace (b), which is obtained upon the decrease of the droplet sizes in the helium-filled octupole cell, are expected to show a more linear laser pulse energy dependence. Finally, the bands in trace (a) appear narrower than those in trace (b). For example, the band at 3610.8 cm^–1^ has a FWHM of 5.5 cm^–1^ in trace (a) and 7.8 cm^–1^ in (b). The signal in trace (b) stems from smaller droplets (yet to be specified) and probably shows some broadening related to the droplet size distribution.

In order to assign the spectra, we performed *ab initio* calculations based on the density functional theory using Gaussian 09.^30^ The IR frequencies and intensities of the (H_2_O)_2_^+^ were obtained at the MPW1K/6-311++G(3df,2p) level of theory,^14^ which was previously benchmarked for (H_2_O)_2_^+^ by comparison with the results from the CCSD(T)/CBS calculations^18^ and many other calculations at similar levels of theory.^31^ The calculated harmonic frequencies were scaled by a factor of 0.928. This scaling factor was chosen as an average of the values determined by a comparison of the measured and calculated frequencies for the PT (0.924) and the Hemi (0.932) cations. Figure 3 shows the calculated structures of the PT-type and the Hemi dimers. Our calculations show that the energy of the PT structure is lower by 36.7 kJ/mol (with zero-point energy correction) than the Hemi structure. In the PT dimer, the H_2_O and OH units are linked by a hydrogen bond sharing a proton. In the metastable (H_2_O·OH_2_)^+^ Hemi dimer, the electron hole is equally shared between two H_2_O units at their lone pairs.^22^

**Figure 3:**
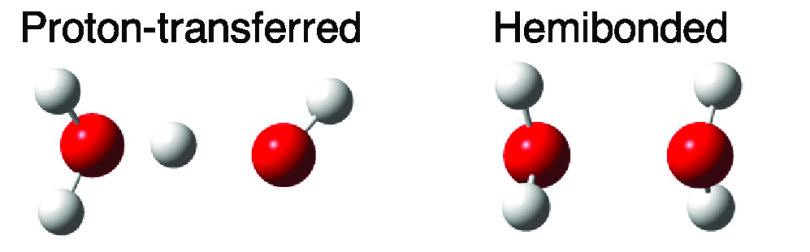
Calculated structure of the PT and Hemi (H_2_O)_2_^+^ dimers.

Figure 4 shows the comparison of the experimental spectrum (black trace, identical with trace (b) in Figure 1) with the spectra calculated for the PT (red) and Hemi (blue) isomers. Each band was convoluted by a Gaussian line shape of the FWHM of 4 cm^–1^. It is seen that the combination of the PT and Hemi calculated spectra well reproduces the measured spectrum. The Hemi dimer has two equivalent H_2_O moieties, and its spectrum has two prominent bands in the OH stretching region (out-of-phase symmetric and in-phase antisymmetric). On the other hand, the spectrum of the PT dimer has four bands: symmetric and antisymmetric free OH stretches of the hydronium moiety, the band of the OH radical unit, and the band due to the hydrogen bond OH (the shared proton).

**Figure 4:**
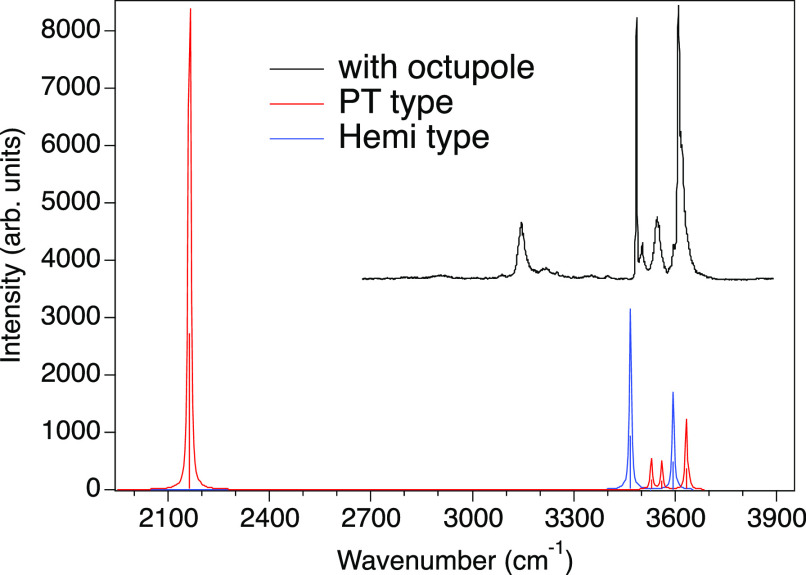
Comparison between the measured (with octupole) and calculated (PT and Hemi types) spectra of (H_2_O)_2_^+^. The calculations were performed at the MPW1K/6-311++G(3df,2p) level. The spectra for the PT and Hemi complexes are shown by red and blue traces, respectively.

The previous IR spectroscopic study of the Ar-tagged (H_2_O)_2_^+^ revealed a strong triplet around 2000 cm^–1^, which was assigned to the vibration of the bridged (shared) proton, in agreement with the results of *ab initio* calculations.^13^ This frequency was beyond the spectral range of the laser system used in this work. Gardenier et al.^13^ assigned the weak band around 3300 cm^–1^ in the Ar-tagged spectrum to the first overtone transition of the shared proton vibration. The large anharmonicity and large IR intensity of the shared proton vibration contribute to the appearance of the overtone band. The calculations in ref (13) also showed that the attachment of the Ar atom leads to the shift of the fundamental shared proton band upward by 100 cm^–1^ and to the weakening of its IR intensity relative to other OH stretch bands by less than one-half. Because of the small effect of the helium environment, the shift is expected to be smaller and the intensity of the overtone larger as compared to those of Ar-tagged ions. Therefore, we assigned the prominent peak observed at 3146.1 cm^–1^ in Figure 1b to the overtone band of the shared proton vibration of the PT dimers in helium droplets. While the three calculated OH stretching bands of the PT dimer disagree with the two strong and sharp bands observed at 3486.6 and 3610.8 cm^–1^, the calculated spectrum of the Hemi dimer has two strong bands, like the measured spectra. Therefore, we assign the 3486.6 cm^–1^ peak to the out-of-phase symmetric stretching band and the 3610.8 cm^–1^ peak to the in-phase antisymmetric stretching band, both in the H_2_O moieties of the Hemi dimer. The three peaks at 3503.8, 3547.0, and 3619.7 cm^–1^ are assigned to the OH stretching bands of the PT dimers. The assignments are summarized in Table 1, which also includes the corresponding calculated frequencies and IR intensities. The assigned peak positions for the Hemi dimer seem reasonable because they lie almost at the middle of the frequencies of the neutral and ionic monomers. A similar pattern was reported for the charge-shared dimers in aromatic dimer cations.^32^

**Table 1:** Measured Frequencies of the Bands of the (H_2_O)_2_^+^ Spectrum and Their Assignments; Scaled Calculated Vibrational Frequencies and IR Intensities at the MPW1K/6-311++G(3df,2p) Level of Theory for PT and Hemi Dimers

experiment (cm^–1^)^a^	configuration type	vibrational mode	calculation (cm^–1^)	intensity (km/mol)
3146.1	PT	overtone of sp (||) shared proton stretch	2164^b^	2728^b^
3486.6	Hemi	out-of-phase symmetric OH stretch	3468	950
	Hemi	in-phase symmetric OH stretch	3527	0.94
3503.8	PT	OH stretch	3530	170
3547.0	PT	symmetric (OH_2_) stretch	3561	147
3596.7^c^	Hemi	out-of-phase antisymmetric OH stretch	3593	28
3610.8	Hemi	in-phase antisymmetric OH stretch	3596	495
3619.7	PT	antisymmetric (OH_2_) stretch	3634	375

a The values are the average from the two spectra when available.

b Fundamental wavenumber and intensity.

c Tentative assignment.

The assignments of the bands are partly supported by the line width. The strong bands at 3486.6 and 3610.8 cm^–1^, assigned to the Hemi dimers, have narrow widths compared to the other bands which were assigned to the PT dimers. The large line width of the bands in the PT dimers may be caused by the efficient relaxation of the free OH stretching modes to the shared proton mode, whose anharmonicity is quite large and is likely to cause a strong coupling to other modes. One may consider an alternative assignment of the 3146.1 cm^–1^ peak to the overtone band of H_2_O bending in the Hemi dimer, though its broad line width is not accounted for.

The pickup pressure dependence in Figure 2 indicates that the (H_2_O)_2_^+^ ions are formed upon ionization of the water dimers in helium droplets. The neutral water dimer has the hydrogen bonded structure in the gas phase^33^ and in helium droplets.^34^ The electron impact ionization of the droplets likely first leads to the formation of He^+^, which is followed by charge transfer to the water dimer and its subsequent ionization. The vertical ionization energies of the dimer were obtained from the photoelectron spectra^35^ to be 12.1 and 13.2 eV, corresponding to the formation of the dimer cation in the low-lying electronic states ^2^A″ and ^2^A′, respectively. Tachikawa^17^ showed that the dynamics of the dimer after the vertical ionization from the neutral structure differs for the two electronic states. The formation of the ground state of the ions ^2^A″ involves the removal of an electron from the proton-donor site, followed by the formation of the PT isomer via the proton transfer to the acceptor unit. On the other hand, the excited state of the (H_2_O)_2_^+^^2^A′ is formed upon the removal of an electron from the proton-acceptor unit. This process breaks the hydrogen bond due to repulsion between the resulting positive charge in the acceptor site and the dipole moment of the proton-donor unit. It induces a rotational motion of the donor unit and allows for the formation of a metastable Hemi isomer with some excess energy. Calculations show that the relaxation of the Hemi to PT structure involves surmounting the energy barrier of about 2.2 kJ/mol.^19^ Therefore, rapid cooling after ionization from the neutral dimer is required for the stabilization of the Hemi dimer. Such a cooling process to local minimum structures was often observed in neutral cluster systems in helium droplets.^36^ This rapid cooling mechanism is lacking in the gas phase, where mostly PT dimers are produced.

It should be noted that the observed intensity ratio in Figure 4 does not directly reflect the abundance of the two isomers. The signal strength depends not only on the IR intensity of the bands but also on the yield of the bare ions upon vibrational excitation of the ions embedded in helium droplets. For the Hemi isomer, the energy of the absorbed IR photon (>3000 cm^–1^) can be consumed to overcome the activation barrier to the PT dimer, causing the release of ∼6000 cm^–1^ upon the isomerization. This additional energy release leads to evaporation of the larger number of He atoms from the droplet and a larger yield of the bare ions. Therefore, the PT dimer may still be the most abundant isomer formed, even in helium droplets.

In summary, we studied the formation of water radical cation dimers by IR spectroscopy in helium nanodroplets. The spectra were assigned to bands of the PT dimers and Hemi dimers, the latter of which has been predicted but not observed previously. The observation of the Hemi dimers deepens microscopic insight into the radiation chemistry of water by providing a clear picture of the hitherto unidentified form of the hydration structure of excess charge. The formation of the Hemi dimer is attributed to the efficient cooling of the incipient ions in the superfluid helium environment. This study demonstrated that helium droplets behave as a cold reaction bath suitable for in situ studies of ion–molecule reactions within molecular clusters. Further studies of larger water cation clusters in helium droplets will provide more information on the mechanism of the hemibond formation and its yield with respect to the clusters containing the proton-transferred motif.
